# Adjuvant radiotherapy and chemoradiation with gemcitabine after R1 resection in patients with pancreatic adenocarcinoma

**DOI:** 10.1186/s12957-015-0560-3

**Published:** 2015-04-15

**Authors:** Daniel Habermehl, Ingo C Brecht, Frank Bergmann, Stefan Rieken, Jens Werner, Markus W Büchler, Christoph Springfeld, Dirk Jäger, Jürgen Debus, Stephanie E Combs

**Affiliations:** Department of Radiation Oncology, Klinikum rechts der Isar, TU München, Ismaninger Str. 22, 81675 Munich, Germany; Department of Radiation Oncology, University Hospital of Heidelberg, Im Neuenheimer Feld 400, 69120 Heidelberg, Germany; Institute of Pathology, University of Heidelberg, Im Neuenheimer Feld 220/221, 69120 Heidelberg, Germany; Department of Visceral Surgery, Klinikum der Universität München (LMU), Marchioninistraße 15, 81377 Munich, Germany; Department of Visceral Surgery, University Hospital of Heidelberg, Im Neuenheimer Feld 110, 69120 Heidelberg, Germany; National Center for Tumor Diseases, Medical Oncology, University Hospital Heidelberg, University of Heidelberg, Im Neuenheimer Feld 460, 69120 Heidelberg, Germany

**Keywords:** Radiotherapy, Pancreatic cancer, Adjuvant radiotherapy, Incomplete resection, R1 resection

## Abstract

**Background:**

The purpose of the study was to evaluate the effect of radiation therapy and chemoradiation with gemcitabine (GEM) after R1 resection in patients with pancreatic adenocarcinoma (PAC).

**Methods:**

We performed a retrospective analysis of 25 patients who were treated with postoperative radiotherapy (RT) or chemoradiation (CRT) after surgery with microscopically positive resection margins for primary pancreatic cancer (PAC). Median age was 60 years (range 34 to 74 years), and there were 17 male and 8 female patients. Fractionated RT was applied with a median dose of 49.6 Gy (range 36 to 54 Gy). Eight patients received additional intraoperative radiotherapy (IORT) with a median dose of 12 Gy.

**Results:**

Median overall survival (mOS) of all treated patients was 22 months (95% confidence interval (CI) 7.9 to 36.1 months) after date of resection and 21.1 months (95% CI 7.6 to 34.6 months) after start of (C)RT. Median progression-free survival (mPFS) was 13.0 months (95% CI 0.93 to 25 months). Grading (G2 *vs*. G3, *P* = 0.005) and gender (female *vs*. male, *P* = 0.01) were significantly correlated with OS. There was a significant difference in mPFS between male and female patients (*P* = 0.008). A total of 11 from 25 patients experienced local tumour progression, and 19 patients were diagnosed with either locoregional or distant failure.

**Conclusions:**

We demonstrated that GEM-based CRT can be applied in analogy to neoadjuvant protocols in the adjuvant setting for PAC patients at high risk for disease recurrence after incomplete resection. Patients undergoing additive CRT have a rather good OS and PFS compared to historical control patient groups.

## Background

Most patients suffering from pancreatic adenocarcinoma (PAC) may not undergo a curative resection because of either locally advanced disease associated with lymph node invasion or disseminated disease [1,2]. Therefore, only about 10% to 20% of all tumours can be resected, and even resected patients show a median survival of 10 to 40 months in newer cohorts [3]. The standard therapy for PAC is a surgical complete resection, but long-term survival of affected patients is still bad. Over the last decades, an improvement of surgical techniques has been stated with a survival advantage for patients undergoing pancreaticoduodenectomy [3]. Even if surgery was well performed with negative resection margins, patients would still have a high risk of locoregional and distant tumour recurrence [4,5]. A recent large monoinstitutional review of patients undergoing adjuvant chemoradiation (CRT) reports a recurrence rate of 87% in all patients, with 55% of all patients experiencing a locoregional relapse (local, peritoneal) [6]. There was no difference in survival between R0- and R1-resected patients. Further reports on the influence of complete and incomplete resections on survival are conflicting. Brennan and colleagues invented a nomogram with risk factors influencing survival after multivariate analysis (tumour size, nodal status, need for splenectomy, grade, tumour localization, posterior margin status) and found a significant dependency of the posterior margin status on survival [7]. In contrast to these findings, Fusai and colleagues found no significant difference in survival of R0- and R1-resected patients [8].

However, adjuvant systemic therapy can be seen as international standard after curative intended surgical resection, whereas combined CRT protocols with gemcitabine (GEM) or fluorouracil (FU; plus folinic acid (FA)) are becoming more widely used in the primary setting for unresectable PAC with encouraging results [9]. An optimal or rather consensus therapy for incomplete resected patients is not observed [6,9]. In case of complete resected PAC, many centres especially in Europe administer adjuvant chemotherapy with FU/FA during a time period of 6 months after surgery according to the large randomized but controversial phase III ESPAC trial [10,11] or with GEM [12]. In the US, there is a trend for combined CRT protocols using FU/FA for the same indication according to the randomized phase III RTOG 97–04 trial [12] and large monoinstitutional analyses [13].

However, patient groups were heterogeneous concerning the resection state (R0, R1), and in most trials and retrospective reviews, there was no difference in survival between both groups [8,14,15]. The feasibility and efficacy of combined therapy regimens consisting of radiotherapy (RT) and GEM were recently demonstrated by the randomized phase III GERCOR trial which examined the outcome of patients after R0 resection with this protocol [16]. According to our institutional policy, patients with R0 resections are treated with FU/FA, whereas R1-resected patients are discussed in an interdisciplinary setting. We have identified a group of 25 patients that underwent CRT with gemcitabine after incomplete resection or who were at high risk for recurrence in our institution during the last decade and present the therapeutic outcome and tolerance of this homogenously treated patient cohort.

## Methods

### Patients and methods

#### Patient characteristics

From 2001 to 2009, a total of 25 patients underwent postoperative RT or CRT after surgical resection of PAC. Each patient was evaluated and discussed in an interdisciplinary setting with experienced surgeons, radiation oncologists and oncologists before treatment initiation. All patient characteristics are shown in Table 1. Patients included in running clinical trials orchestrated by our centre were excluded from this analysis, and results are and will be presented elsewhere.

**Table 1:** **Patient characteristics**

**Patient characteristics**	**Values**
Number	25
Gender	
Male	17 (68%)
Female	8 (32%)
Age (years)	
Median, range	60 (34 to 74)
Localization	
Head	21
Body and/or tail	4
Lymph node status	
Positive	16 (64%)
Negative	9 (36%)
Grading	
G1	0
G2	11
G3	11
G4	1
Not reported	2^a^
RTX	
EBRT median dose (range) (n = 26)	49.6 Gy (36 to 54 Gy) RT
IORT median dose (range) (n = 7)	IORT: 12 Gy (10 to 15 Gy)
Concomitant CTX	
Overall (with gemcitabine)	23 (92%)
None	2 (8%)
Toxicity	
Gastrointestinal (I to III°)	17
Haematological (I° to IV°)	17
Grade II	11
Grade III	5
Grade IV	1
Previous tumour resection	None
Duration resection - onset of RT	
Median, range [days]	44 (12 to 100)
<30 days	5
30 to 50 days	11
>50 days	9

RT radiotherapy, Gy grey, IORT intraoperative radiotherapy, CTX chemotherapy, EBRT external beam radiotherapy. ^a^Explanation in ‘Patients and methods’ section.

#### Surgery and histopathological diagnosis

Evaluation of the histopathological resection status, TNM status as well as AJCC staging and histological grading was performed according to the criteria recommended by the WHO (2010) [17]. All histological slices were re-evaluated for this analysis by a pathologist with high expertise in that field. Resection margins were prepared and analysed according to a detailed pathological procedure described by Esposito *et al*. [18]. Therefore, R1 was defined when the distance of the tumour from the resection margin was less or equal 1 mm.

#### Radiation and chemotherapy

For treatment planning, CT imaging was performed according to an in-house standard protocol in supine position. 3-D treatment planning was performed in 22 patients and intensity-modulated radiation therapy (IMRT) using either step-and-shoot or helical (tomotherapy) radiotherapy in 3 patients, defining two treatment areas: Clinical target volume (CTV) included the initial pre-operative macroscopic tumour extension and relevant regional lymph node areas (elective nodal irradiation (ENI)). For the planning target volume (PTV), a safety margin of approximately 10 to 15 mm was added to the CTV and was dependent on setup accuracy and respiratory motion. Median PTV volume was 497 ccm (range 223 to 1,400 ccm). In 11 patients, an additional boost volume was defined if appropriate and included the area of initial macroscopic tumour extension with a small safety margin (PTV_boost). Median PTV_boost volume was 98 ccm (range 38 to 260 ccm).

None of the analysed patients had a history of abdominal RT. One patient (3%) had a history of previous chemotherapy with gemcitabine (GEM) before surgery. Ninety-two percent of all patients undergoing RT received concurrent chemotherapy with GEM with a dose of 300 mg/m^2^ weekly, followed by adjuvant cycles of full-dose GEM (1,000 mg/m^2^). Additionally, 14 patients (54%) received full-dose GEM after completion of CRT. Before application of GEM, blood values were examined at the day of treatment or 1 day before and leucocytes had to be ≥3,000/μl and platelets ≥100,000/μl. Furthermore, physical examination was performed, and there had to be no evidence of severe infection. During combined CRT and during the adjuvant cycles, blood cell count and a physical examination were conducted weekly.

Fractionated RT was applied with a median dose of 49.9 Gy (range 36 to 54 Gy) in median single fractions of 1.8 Gy, including dose for image guidance (IGRT). Seven patients received additional intraoperative radiotherapy (IORT) with a median dose of 12 Gy (range 10 to 15 Gy) during surgical resection. In these cases, indication for IORT was decided by the surgical team and was usually performed if resection margins were expected to be positive for residual tumour tissue. However, this was left to the discretion of the treating surgeon and depended on individual patient and tumour characteristics during resection. Patients that previously underwent IORT received a lower median external beam radiotherapy (EBRT) dose of 41.4 Gy compared to patients without IORT (median dose 50.4 Gy).

#### Follow-up and statistics

Four to 6 weeks after completion of chemoradiation and after 1 cycle of full-dose GEM evaluation of treatment response and assessment of resectability were performed. In most patients, contrast-enhanced CT or MR imaging was conducted. Additionally, follow-up visits including clinical examination as well as tumour marker analysis were performed. The images as well as the clinical performance status of the patients were evaluated.

Overall survival was calculated from the first day of irradiation until death. Progression-free survival (PFS) was calculated from the first day of radiotherapy until progression of disease (local or distant metastasis). The log-rank test was implemented to compare survival curves evaluating the association between clinical variables of interest and survival. All calculations were performed using statistical software program SPSS 18.0 for Windows (Chicago, Illinois, USA). The comparison to historical data was drawn from data published in the literature, no formal comparison of Kaplan-Meier data was performed.

## Results

### Survival

**Figure 1:**
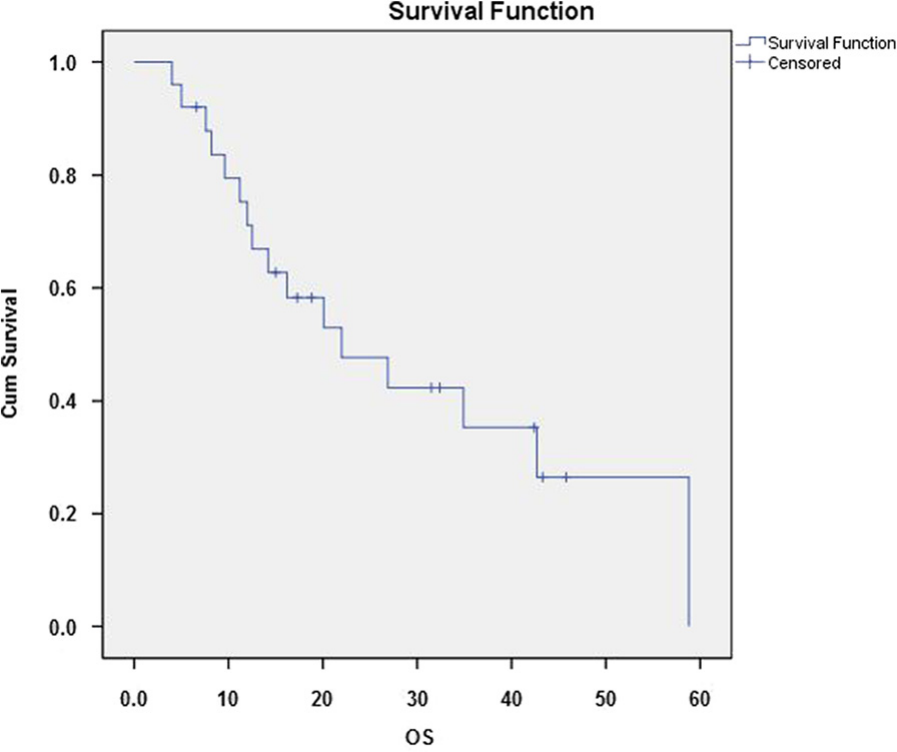
Kaplan-Meier curve representing patients’ overall survival.

Median age was 60 years (range 34 to 74 years), and there were 17 male (68%) and 8 (32%) female patients. Median overall survival (mOS) of all treated patients was 22 months (95% confidence interval (CI) 7.9 to 36.1 months) after date of resection and 21.1 months (95% CI 7.6 to 34.6 months) after start of CRT (Figure 1). Potential prognostic factors such as applied IORT (*P* = 0.85), dose of fractionated RT (dose ≤45 *vs*. >45 Gy, *P* = 0.08), duration from date of surgery until first day of CRT (<30 days, 30 to 50 day, >50 days, *P* > 0.05), positive resected lymph nodes (*P* = 0.89) and localization (pancreatic head *vs*. body, *P* = 0.58) and age (≤55 *vs*. >55 years; *P* = 0.35) were evaluated. None of these parameters had a significant influence on OS, only grading (G2 *vs*. G3, *P* = 0.005) and gender (female *vs*. male, *P* = 0.01) were significantly correlated with OS (Figure 1).

### Progression-free survival and patterns of relapse

Main localization of the tumour was the pancreatic head or body and tail in 21 (84%) and 4 (16%). Median time from surgery until start of CRT was 41 days (range 12 to 100 days). Positive lymph nodes were diagnosed in 16 patients (64%) whereas 9 (36%) patients had negative nodes. Histopathological grading was classified G2, G3 and G4 in 11 (44%), 11 (44%) and 1 (4%) patients, respectively. In 2 patients (8%), no information on grading was achievable due to missing information from external institutions.

**Figure 2:**
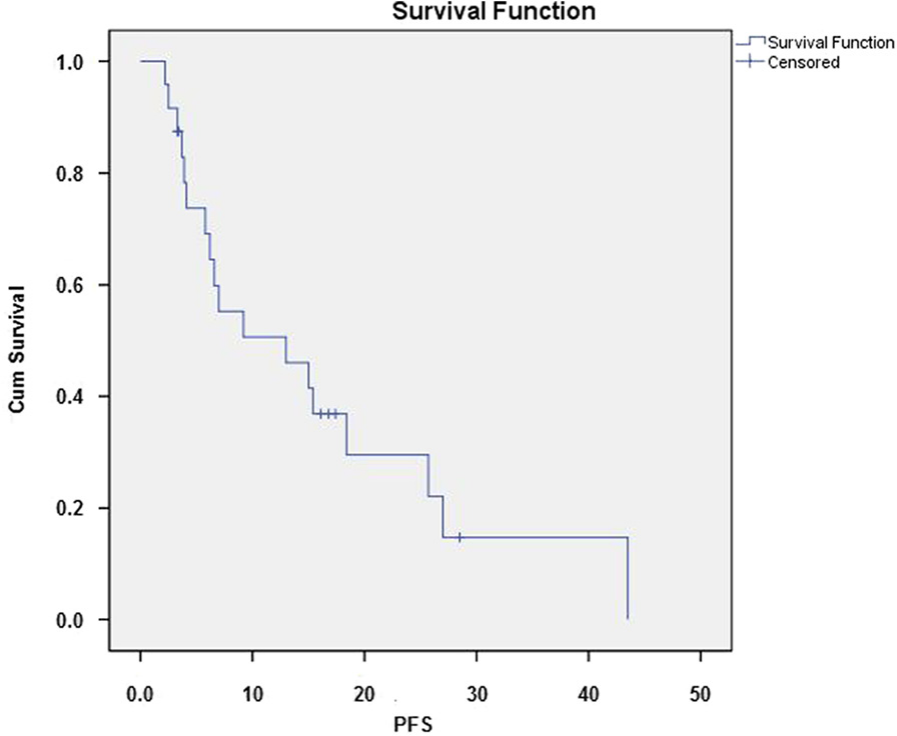
Kaplan-Meier curve for patients’ progression-free survival.

Median progression-free survival was 13.0 months (95% CI 0.93 to 25 months) (Figure 2). No significant correlation was found between PFS and IORT (*P* = 0.85), tumour localization (*P* = 0.7), grading (*P* = 0.3), nodal status (0.4), duration between resection and RT onset (*P* = 0.9), age (*P* = 0.8) and dose (≤45 *vs*. >45 Gy) (*P* = 0.2). There was a significant difference in median time of progression-free survival between male and female patients (0.008), in favour of women.

**Figure 3:**
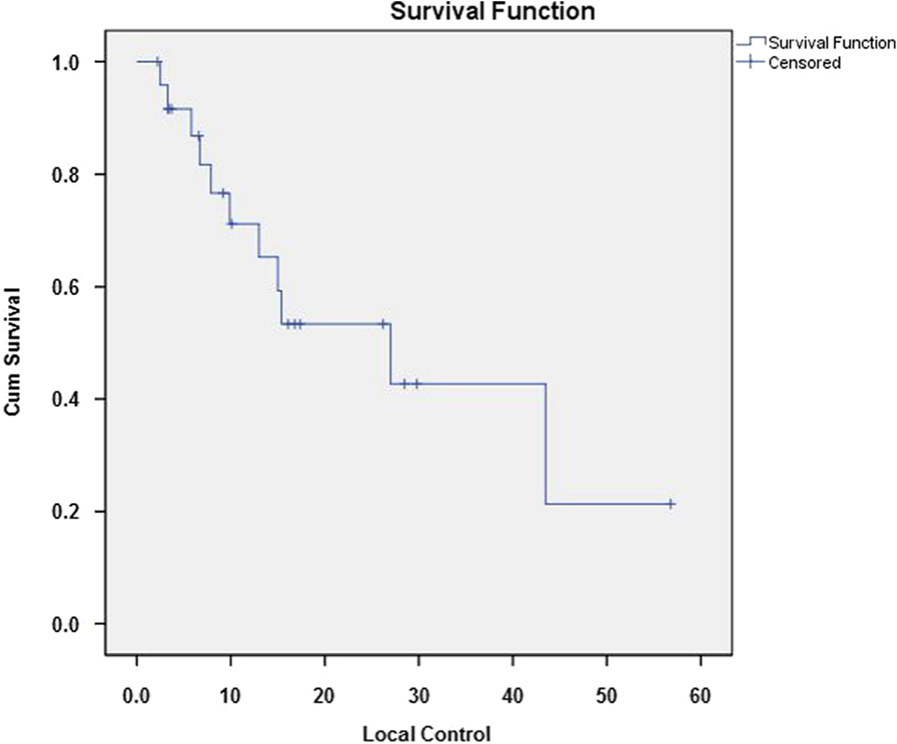
Kaplan-Meier curve for patients’ local control.

Median local control according to Kaplan-Meier estimates was 27 months (95% CI 8.1 to 45.9 months) since start of combined or single modality (C)RT (Figure 3). A total of 11 from 25 patients (44%) experienced local tumour progression. Analysis of potentially associated treatment-related factors showed no influence of IORT (*P* = 0.78), patient age (*P* = 0.96), EBRT (*P* = 0.1), tumour localization (*P* = 0.7), tumour grading (*P* = 0.09), presence of positive lymph nodes (*P* = 0.5) and duration from surgery until RT onset (*P* = 0.2) on local disease control. Again, female gender was correlated with a higher local control (*P* = 0.02).

Nineteen patients (76%) were diagnosed with either locoregional or distant failure over the course of their follow-up. Main sites of distant disease progression were the liver, peritoneum, lungs and bone in 10 (40%), 4 (16%), 1 (4%) and 1 (4%) patients, respectively.

### Toxicity

In general, observed side effects were mild, and thus, no treatment-related deaths occurred. Seventeen patients had moderate gastrointestinal toxicity (grade I to III), mainly consisting of nausea, vomiting, diarrhoea and abdominal pain. Haematological toxicity consisted mainly of grade II (12/25 patients, 48%) but also of grade III in 5 (20%) and grade IV in 1 patient (4%). The side effects could be treated successfully by supportive care.

## Discussion

In our patient cohort with R1 resection and postoperative administration of GEM-based CRT, mOS of 22 months postresection could be achieved. The median time to disease progression was 13 months and consisted mainly of local progression (44%) and metastasis to the liver (40%). Nine patients with a local progression had a median time of 9.9 months until imaging- or surgical-based evidence of tumour relapse. Median time of local control of all included patients was comparably high with estimated 27 months but has limited significance because less than half of the patients developed local recurrence. Nevertheless, there are a considerable high number of patients without a local tumour progression after 12 months (12 patients) and even after 2 years (6 patients).

During the last years, one large randomized clinical trial on adjuvant radiotherapy after R0 and R1 resections was performed at our high-volume centre: results were recently published [19]. Many patients were therefore included in this trial. Moreover, a considerable number of patients have not received any adjuvant or additive treatment or were treated with systemic agents (for example, gemcitabine, 5-FU). This may explain the comparable low number of patients included in our current analysis. Due to the scarce data in the literature on adjuvant RT and CRT, the current analysis seems justified.

Survival data are difficult to compare to the outcome of the abovementioned large randomized clinical trials which mainly included patients after R0 resection but also patients with positive margins, for example, approximately 17% of patients in the CONKO-001 trial [20], 18% in the ESPAC trial [15] and 35% in the Johns-Hopkins-Mayo collaborative experience [13]. A further meta-analysis of five published randomized clinical trials (RCT) showed a range of ‘margin positive’ resections of 0% to 83% with a median of 23% [14]. Compared to the reported overall outcome of the mentioned trials as well as to the subgroups of R1/R2-resected patients, the mOS achieved in this study (with a standardized histopathological protocol) seems to be at least equieffective. The CONKO-001 group reports a R1 subgroup-specific mOS of 22.1 months (GEM group and R1-resected), the ESPAC trialists present an mOS of 15.9 in the CRT group and the bi-institutional study from Hsu and colleagues reports a mOS of 15.1 months for R1/R2-resected patients after CRT. The meta-analysis of Butturini *et al*. revealed a mOS of 14.7 months for the subgroup of patients with R1 resection and after adjuvant CRT.

Long-term survival after curative intended resection for PAC has been described by a recent case–control study from the Mayo Clinic [21]. Schnelldorfer *et al*. analysed 357 patients, of which a total of 62 (17%) survived at least 5 years and further 21 (6%) were alive even 10 years after treatment. The median survival times were 18, 15 and 10 months after R0, R1 and R2 resections, respectively. A multivariate analysis on prognostic factors showed that resection margin is not an independent prognostic factor. In our analysis, we found 9 patients (36%) that were still alive for at least 2 years, taking into account a comparable short mean follow-up period of 21 months.

Until now, there exists no international standardized adjuvant or additive therapy after resection of PAC. European institutions tend to use postoperative chemotherapy protocols mainly containing full-dose GEM for six cycles mainly based on the large randomized CONKO-001 trial [20] or FU/FA as tested in the ESPAC trials [11,15] whereas CRT protocols including FU/FA are rather established in the US according to large monoinstitutional experiences [13]. The approach of our institution in patients with residual disease or at high risk of tumour recurrence is a GEM-containing CRT which has proven efficacy in neoadjuvant, recurrent and adjuvant biliary and pancreatic disease [4,9,22]. Recent approaches have also evaluated the potential benefit of a more aggressive systemic agent regime including interferon (IFN) α-2b [19]. For this purpose, 132 R0/R1-resected patients received either FU, cisplatin, and IFN α-2b concomitant with RT followed by two cycles of FU (arm A, *n* = 64) or six cycles of FU monotherapy (arm B, *n* = 68). Median survival for all randomly assigned patients was comparable in both arms (26.5 and 28.5 months in arms A and B, respectively). Although median survival was very good in both arms, substantial therapy-related higher toxicities grades III and IV were recorded in as many as 85% and 16% in arms A and B, respectively.

Defining the R1 resection status as well as its significance on survival is still a matter of debate among specialists. Patients recruited in the abovementioned large trials were usually not stratified according to the extent of resection and clearly vary in the number of R1 resections [14]. The significance of clear resection margins compared to R1 resections was indeed emphasized by different groups, but other groups failed to find differences in survival times between R0- and R1-resected patients, or difference was small [8,23,24]. A possible explanation of these conflicting findings is a difference in the definition of an R1 resection, and thus, the pathological assessment of the specimen after pancreaticoduodenectomy which significantly varies among centres and mirrors a lack of international consensus [25,26]. Whereas some centres define the R1 resection according to the ‘old’ rule that microscopic tumour cells must invade the resection border, others classify even a distance up to 1 mm to the border as R1 [25]. As a consequence of this high variability in resection margin definition among centres, it has to be assumed that R1 resections are clearly underrepresented in published studies according to Verbeke and Chang *et al*. [25,27]. Recently, an important approach on that issue was undertaken by Chang and co-workers who examined in detail the relationship between margin clearance and outcome in 365 PAC patients after curative-intended surgery [27]. Patients were divided into different subgroups depending on the extend of tumour resection/margin clearance (0 to 0.5 mm, 0.5 to 1 mm, 1 to 1.5 mm and greater than 1.5 mm). Influence of the margin clearance on locoregional disease recurrence, median survival and especially long-term survival was analysed. Surprisingly, there was no difference detected in mOS between patients with a directly involved resection margin and those who had a margin clearance of more than 1.5 mm, but long-term survival was better in the latter group. Finally, patients after R1 resection are at different risk levels for disease recurrence or disease progression. Whereas all PAC patients are at high risk to have an occult metastatic disease, there may be a higher risk for locoregional recurrence/progression in patients with a higher postsurgical tumour load depending on margin clearance, thus advocating a local radiotherapy. Patients with clear margins have a relatively higher risk of developing distant metastatic disease and may therefore benefit more from a systemic chemotherapy. This has been shown in a meta-analysis of Stocken *et al*. where patients with R0 resection had the most benefit from adjuvant chemotherapy compared to patients with residual microscopic tumour residues [28]. According to these findings, one may hypothesize that patients with a closer resection of 0.5 mm have a higher probability to benefit from an intensified adjuvant local treatment such as CRT. In the light of the comparably superior outcome of our R1-resected patient cohort, one may hypothesize that many of the included specimens would be classified as R0 in other centres with less strict protocols with the corresponding implication of improved survival times.

Female gender was positively correlated with PFS and OS in our analysis. Recent reports on prognostic factors of PAC patients undergoing adjuvant or additive treatment are differing. Population-based and other large retrospective analyses failed to identify gender as a prognostic factor in patient groups undergoing adjuvant treatments [13,29,30]. However, Redmond and co-workers from Johns Hopkins University found that female gender has prognostical benefit in terms of improved OS in a monoinstitutional analysis of patients undergoing adjuvant 5FU-based CRT [31].

GEM-based regimens become more widely used in hepatobiliary and pancreatic cancer because of its efficacy and good tolerability [4,9,16,22,32,33]. Toxicity rates were comparable to data derived from larger reports and clinical trials. We observed a grade III or higher haematological toxicity in 24% of all patients. This percentage is comparable to the ESPAC-3 study where 22% of the patients in the FU + FA and the GEM group experienced grade III/IV toxicities (neutrophil count) and the GERCOR study where 33% of the patients in the combined GEM-containing CRT group experienced grade III/IV toxicity (neutrophil count) [16,34].

Our retrospective analysis is the first report to our knowledge on adjuvant GEM-based CRT for PAC patients after R1 resection, with the latter being confirmed by a standardized pathological procedure [18]. The study has mentionable limitations, mainly based on a low number of patients included, the non-randomized character as well as the difficult comparison to heterogeneous historical collectives. However, considering the few publications in this clinical setting and the difficult decision-making in individual clinical situations even in high-volume centres, the data can be cited as useful treatment recommendation in certain clinical situations. GEM-based CRT is effective and feasible and can be applied in analogy to neoadjuvant protocols at our institution [4,9]. Survival was surprisingly good and nearly a third of all patients being still alive after more than 24 months.

According to international guidelines, RT is no standard adjuvant treatment after resection but is chemotherapy. However, after interdisciplinary discussion, indication for adjuvant CRT is decided upon in certain cases. Further randomized clinical trials are needed to identify patients that benefit most from an intensified local and systemic therapy in the postoperative setting.

## Conclusions

This work demonstrates that GEM-based CRT can be applied in analogy to neoadjuvant protocols in the adjuvant setting for PAC patients at high risk for disease recurrence after incomplete resection. Overall survival and progression-free survival times are considerably high compared to historical control patient groups.
